# Analysis of the efficacy and safety of immunotherapy in advanced thymoma patients

**DOI:** 10.1002/cam4.5357

**Published:** 2022-11-16

**Authors:** Yue Hao, Gen Lin, Jing Xiang, Wenxian Wang, Chunwei Xu, Qian Wang, Jing Cai, Yongchang Zhang, Zhengbo Song

**Affiliations:** ^1^ The Second Clinical Medical College of Zhejiang Chinese Medical University Hangzhou China; ^2^ Department of Medical Oncology The Cancer Hospital of the University of Chinese Academy of Sciences (Zhejiang Cancer Hospital) Hangzhou China; ^3^ Department of Thoracic Oncology, Fujian Medical University Cancer Hospital Fujian Cancer Hospital Fuzhou China; ^4^ Department of Respiratory Medicine, Jinling Hospital Nanjing University School of Medicine Nanjing China; ^5^ Department of Respiratory Medicine, Affiliated Hospital of Nanjing University of Chinese Medicine Jiangsu Province Hospital of Chinese Medicine Nanjing China; ^6^ Department of Oncology Second Affiliated Hospital of Nanchang University Nanchang China; ^7^ Department of Medical Oncology, Lung Cancer and Gastrointestinal Unit Hunan Cancer Hospital/The Affiliated Cancer Hospital of Xiangya School of Medicine, Central South University Changsha China

**Keywords:** efficacy, immune‐related adverse events, immunotherapy, thymoma

## Abstract

**Background:**

Immunotherapy has exhibited efficacy in thymic carcinoma patients; however, there are insufficient data to confirm this efficacy in thymoma. The toxicity of immunotherapy also remains to be determined.

**Methods:**

The efficacy and safety of immunotherapy were analyzed in 11 thymoma patients who received PD‐1 inhibitors according to a range of relevant indexes including the objective response rate (ORR), disease control rate (DCR), progression‐free survival (PFS), overall survival (OS), and immunotherapy‐related adverse events.

**Results:**

The PFS and OS rates for all patients were 12.8 and 56.5 months, respectively. No difference in efficacy was detected between monotherapy and combination therapy (PFS: 12.8 vs 2.2 months, P = 0.787; OS: 73.8 vs 56.5 months, P = 0.367). The ORRs and DCRs for all patients were 27.3% and 90.9%, respectively. The incidence of adverse events was 45.5% among the 11 thymoma patients, including immune‐related myocarditis (36.4%), immune‐related liver damage (18.2%), and myasthenia gravis (18.2%). In the whole cohort of patients, the rate of adverse events of grade 3 or higher was 36.4%. The rates of adverse events of grade 3 or 4 in B3‐type and non‐B3‐type thymoma patients were 0% and 62.5%, respectively.

**Conclusions:**

Immunotherapy elicited a response in thymoma patients; however, more attention should be paid to the immune‐related adverse events.

## Graphical abstract

In our study, efficacy and safety of immunotherapy were analysed in 11 thymoma patients received PD‐1 inhibitors. The PFS and OS rates for all patients were 12.8 and 56.5 months, respectively. However, more attention should be paid to the immune‐related adverse events.

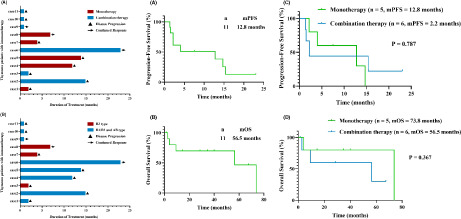

## 1 INTRODUCTION

Thymoma is a rare type of tumor that originates from thymic epithelial cells.^1^ For thymoma patients, there is a reported risk of relapse or metastasis,^2^ so the prognosis tends to be poor.

Therapeutic regimens for thymoma patients depend on the stage of the disease. Regarding chemotherapy, anthracycline‐based chemotherapy shows an improved tumor response rate compared with etoposide.^3^ The CAP regimen, which includes cyclophosphamide, doxorubicin, and cisplatin, has been recommended as a standard first‐line therapy.^4^ However, detailed analyses of therapeutic schemes for thymoma patients are limited and the heart‐related toxicity of the CAP regimen has limited its use.^4^, ^5^ Other targeted therapies, such as sunitinib, also exhibit a limited response rate so immunotherapy has been proposed as a possible alternative therapy. Immunotherapy has exhibited efficacy against some tumor types, for example, it has been approved as the first‐line therapy for non‐small cell lung cancer.^6^ Immunotherapy therefore provides a possible alternative for the treatment of thymoma patients.

A few studies have reported the efficacy of immunotherapy in thymic carcinoma,^7^, ^8^, ^9^ but the outcomes relating to different immunotherapy regimens in advanced thymoma patients have not been determined. The current study aimed to analyze the efficacy and safety of immunotherapy in a cohort of thymoma patients.

## 2 MATERIALS AND METHODS

### 2.1 Patient selection

The study cohort consisted of 11 thymoma patients who received immunotherapy at Zhejiang Cancer Hospital, Hunan Provincial Tumor Hospital, or Fujian Provincial Tumor Hospital between 2019 and 2022. The patients had been diagnosed with advanced thymoma and had undergone immunotherapy with varying therapeutic schedules, that is, monotherapy or combination therapy. All patients had stage IV tumors. Our research was conducted in accordance with the Declaration of Helsinki and individual consent for this retrospective analysis was waived. The protocol was approved by the Institutional Ethics Committee at Zhejiang Cancer Hospital and each investigation site.

### 2.2 Treatment and response assessments

The two majority therapeutic regimens involved monotherapy and combination therapy. The monotherapy regimen involved the administration of a PD‐1 inhibitor, whereas combination therapy regimens involved the administration of an immunotherapy drug combined with paclitaxel or nab‐paclitaxel plus platinum, apatinib, or pemetrexed plus bevacizumab. After two cycles of therapy, disease assessment was accomplished by a CT scan. In the event of immune therapy‐related adverse events (irAEs) or severe disease progression, further CT scans may be performed.

The overall response rate (ORR) was confirmed, along with the complete response (CR) and/or partial response (PR) rate. Progression‐free survival (PFS) was assessed from the first day of immunotherapy to the earliest signs of disease progression or death from any cause. Overall survival (OS) was determined from the date of initiation of immunotherapy to death or the last follow‐up evaluation.

Safety was assessed according to National Cancer Institute Common Terminology Criteria (NCI‐CTC) for Adverse Events, Version 4.03. The grade of irAEs ranged from 1 to 5.

### 2.3 Statistical analysis

The differences between patient characteristics were analyzed by Fisher's exact test using the Bonferroni method. Kaplan–Meier estimates and the log‐rank test were used to evaluate PFS and OS, respectively. All statistical analyses were performed using SPSS (version 25.0; SPSS, Inc.) and GraphPad prism (version 9). The two‐sided *p* value of our study sample was included in analyses, and *p* values were determined by a log‐rank test. Two‐sided *p* values < 0.05 were considered statistically significant. The last follow‐up date was January 15, 2022.

## 3 RESULTS

### 3.1 Patient characteristics

Between January 2019 and January 2022, a total of 11 patients were enrolled in our study and were included in the efficacy and safety analyses. All of the thymoma patients received PD‐1 inhibitors: five patients received monotherapy and six patients received combination therapy.

The baseline characteristics of the patients are summarized in Table 1. The median age of all thymoma patients was 45 years (range, 22–57 years). All patients had stage IV tumors. The ECOG PS (Eastern Oncology Group Physical Status Score) of all of the patients was 0–1. Histologic examination of the tumors revealed the following subtypes: B1/B2, B1, B2, B3, and AB. The B3 subtype was most common, accounting for 27.3% of patient tumors. As for therapy, seven patients received PD‐1 inhibitors as the first‐line therapy, two patients received PD‐1 inhibitors as the second‐line therapy, and two patients received immunotherapy as the third‐line therapy.

**TABLE 1:** Clinicopathological characteristics of thymoma patients who received immunotherapy

Case	Gender/age	Stage	Smoking	Histology	Surgery	Radiotherapy	PFS	OS	Therapy mode	Response	Therapy line
1	Male/51	IV	No	B1 + B2	Yes	No	2.2	73.8	monotherapy	PD	First line
2	Female/50	IV	No	B2	Yes	No	15.4	28.6	combination	PR	First line
3	Female/47	IV	No	B3	Yes	Yes	2.2	56.5	combination	SD	First line
4	Male/34	IV	No	B2	Yes	No	12.8	34.7	monotherapy	SD	First line
5	Female/57	IV	Yes	B2	No	No	14.7	40	monotherapy	SD	Second line
6	Female/54	IV	Yes	B1	No	Yes	23	67.5	combination	PR	Second line
7	Female/42	IV	Yes	B3	No	Yes	4	4	monotherapy	SD	Third line
8	Female/42	IV	Yes	B3	No	No	7.5	14.5	monotherapy	SD	First line
9	Female/22	IV	Yes	AB	No	No	1.5	1.5	combination	PR	First line
10	Male/45	IV	No	AB	No	No	1.4	2.7	combination	SD	First line
11	Male/45	IV	Yes	B1 + B2	Yes	Yes	1.5	9.3	combination	SD	Third line

Abbreviations: OS, overall survival; PD, progressive disease; PFS, progression‐free survival; PR, partial response; SD, stable disease; Therapy mode (including monotherapy and combination therapy); Time (months).

### 3.2 Treatment response and survival analysis

**FIGURE 1:**
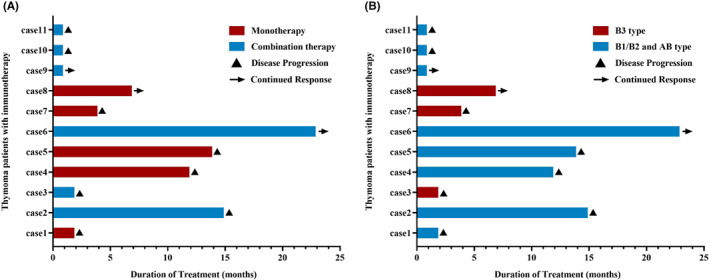
A swimmer's plot showing the time to treatment discontinuation for all thymoma patients who received PD‐1 inhibitors. (A) The difference between monotherapy and combination therapy; (B) the difference between B3 and non‐B3‐type thymoma

**FIGURE 2:**
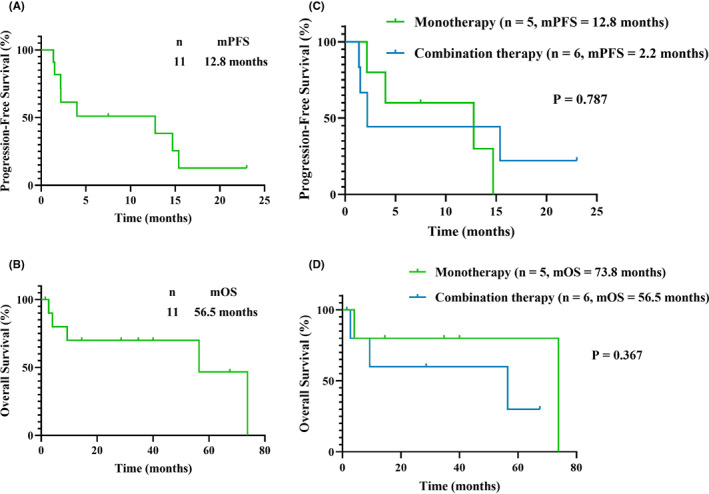
Kaplan–Meier estimates of progression‐free survival (PFS) and overall survival (OS). (A) PFS of the whole cohort of thymoma patients (*n* = 11, PFS = 12.8 months); (B) OS of the whole cohort of thymoma patients (*n* = 11, OS = 56.5 months); (C) the difference in PFS between monotherapy and combination therapy for thymoma patients (12.8 vs. 2.2 months, *p* = 0.787); (D) the difference in OS between monotherapy and combination therapy for thymoma patients (73.8 vs. 56.5 months, *p* = 0.367)

An excellent treatment response was obtained in three of the thymoma patients. The ORR of immunotherapy was 27.3%, and the disease control rate was 90.9%. The treatment regimens and analyses of the treatment responses are presented in Figure 1 (panels A and B). The durations of PFS and OS of the thymoma patients who received immunotherapy were 12.8 and 56.5 months, respectively, as shown in Figure 2 (panels A and B). No difference in efficacy was detected between monotherapy and combination therapy (PFS: 12.8 vs. 2.2 months, *p* = 0.787; OS: 73.8 vs. 56.5 months, *p* = 0.367), as shown in Figure 2 (panels C and D), and no significant difference in survival between non‐B3‐type thymoma and B3‐type thymoma patients was detected (PFS: 12.8 vs. 4.0 months, *p* = 0.783; OS: 73.8 vs. 56.5 months, *p* = 0.393). Also, the efficacy of first‐line immunotherapy for thymoma patients had not manifested the difference with the latter‐line therapy (PFS: 12.8 vs. 4.0 months, *p* = 0.597; OS: 56.5 vs. 9.3 months, *p* = 0.645).

### 3.3 Toxicity evaluation

All patients were assessed for toxicity resulting from treatment. The incidence of irAEs was 45.5% among the 11 thymoma patients and events included immune‐related myocarditis (36.4%), immune‐related liver damage (18.2%), and myasthenia gravis (18.2%). Among the whole cohort of patients, the rate of irAEs of grade 3 or higher was 36.4%, which was consistent with the rate of irAEs in non‐B3 thymoma patients. We found that irAEs of grade 3 or higher did not exist in B3‐type thymoma patients. Four patients suffered from immune‐related myocarditis of grade 2 and 3. One patient displayed increasing levels of creatine kinase and reached the criteria for acute myocardial injury, which is a life‐threatening complication that cannot be reversed immediately. Another patient suffered from liver dysfunction of grade 3. Events resulting from toxicity were dyspnea, chest stuffiness, drooped eyelids, muscle pain, and weakness of grade 1. The details are shown in Table 2.

**TABLE 2:** Toxicity experienced by five thymoma patients who received immunotherapy

Case	Type	Myocarditis	Hepatotoxicity	Myasthenia gravis	Other toxicities
Case 1	B1 + B2	Yes	No	Yes	Chest tightness
Case 2	B2	No	Yes	Yes	Fatigue
Case 4	B2	Yes	No	No	Creatine kinase↑
Case 10	AB	Yes	Yes	No	Lung infection
Case 11	B1 + B2	Yes	No	No	None

*Note*: National Cancer Institute Common Terminology Criteria for Adverse Events.

## 4 DISCUSSION

Our study investigated the efficacy of immunotherapy for thymoma patients. It is the first study to comprehensively describe the condition of thymoma patients that received different immunotherapy regimens and to analyze the efficacy and toxicity associated with treatment. These immunotherapy regimens provided new options for the treatment of thymoma patients; however, the high rate of irAEs requires attention.

A previous report described a patient who received one cycle of pembrolizumab monotherapy and maintained an immune response for 9 months after the discontinuation of therapy.^10^ In another case report, a B2‐type thymoma patient who received pembrolizumab monotherapy, maintained an immune response for 2 months.^11^ In our study, the OS of thymoma patients who received immunotherapy was 56.5 months and the PFS was 12.8 months. High efficacy was detected for immune monotherapy, with an OS duration of 73.8 months.

Although the efficacy of immunotherapy was confirmed in thymoma patients, the toxicity of immunotherapy could not be ignored. According to our study, the incidence of irAEs was 45.5% in the 11 thymoma patients, including those with immune‐related myocarditis, for which the incidence was 36.4%. One thymoma patient suffered myocarditis resulting from acute myocardial injury, which was life‐threatening. We observed a phenomenon by which an increasing grade of thymoma was accompanied by fewer irAEs. This was demonstrated by the higher occurrence of irAEs in patients with B1/B2 and AB‐type thymoma. We speculated that higher efficacy of immunotherapy was associated with more severe toxicity in some types of thymoma. Similarly, Song et al. reported that more effective therapy was accompanied by a higher risk of irAEs in patients.^1^ Taken together, these findings indicate that closer attention should be paid to irAEs, especially for B1/B2 and AB‐type thymoma patients. According to a previous study,^10^ one patient who received pembrolizumab and achieved a partial response after two doses, experienced irAEs of grade 3 or lower, which included liver and kidney dysfunction, hypothyroidism, and myocarditis. The use of avelumab has been linked to grade 3 and 4 irAEs in 38% of patients, with all experiencing an autoimmune disorder.^12^ The occurrence of severe irAEs has been linked with nivolumab treatment, even after a single dose, with some conditions persisting even after the cessation of therapy.^13^

Further studies^14^, ^15^ reported higher grade irAEs, including myocarditis and myasthenia gravis, in thymoma patients than in patients with other cancer types. One report^10^ recommended a regimen involving a half‐dose of immunotherapy drug to achieve remission of the tumor, whilst incurring only mild irAEs. Such a strategy may reduce, to some extent, the occurrence of lethal myocarditis, and such regimens should therefore be considered to minimize irAEs.^16^ By reducing the initial dose, one study reported that 55% of irAEs were less severe than with the full dose of therapy.^17^ The use of immunosuppressive drugs may also be worth considering for thymoma patients suffering from irAEs.^18^ In our study, one patient received infliximab and γ‐globulin to effectively overcome myocarditis that occurred after immunotherapy. Although immune‐checkpoint inhibitors are not part of the standard treatment regimen for thymoma patients,^19^ their role in immunotherapy is significant. Future large‐scale clinical trials are needed to explore the benefits of immunotherapy.

This study had some limitations. The small sample size needed to be taken into consideration when interpreting the results, as did the heterogeneity among different subtypes of thymoma. Further research is thereby needed to verify the outcomes.

## 5 CONCLUSIONS

Thymoma patients may benefit from immunotherapy; however, attention should be paid to the possibility of irAEs, particularly lethal myocarditis. In the future, a prospective study is needed to evaluate the efficacy and safety of immunotherapy in advanced thymoma patients.

## AUTHOR CONTRIBUTIONS

**Yue Hao:** Data curation (equal); formal analysis (equal); methodology (equal); software (equal); writing – original draft (lead); writing – review and editing (equal). **Gen Lin:** Data curation (equal); investigation (equal); software (equal); writing – original draft (equal). **Jing Xiang:** Data curation (equal); software (equal); writing – original draft (equal). **Wenxian Wang:** Formal analysis (equal); project administration (equal); visualization (equal); writing – review and editing (equal). **Chunwei Xu:** Methodology (equal); project administration (equal); resources (equal); visualization (equal). **Qian Wang:** Data curation (equal); formal analysis (equal); investigation (equal); software (equal). **Jing Cai:** Data curation (equal); investigation (equal); visualization (equal). **Yongchang Zhang:** Conceptualization (equal); formal analysis (equal); project administration (equal); supervision (equal); writing – review and editing (equal). **Zhengbo Song:** Conceptualization (equal); funding acquisition (equal); project administration (equal); resources (equal); supervision (equal); writing – review and editing (lead).

## FUNDING INFORMATION

The study was granted by the Foundation of CSCO‐Shiyao (Y‐SY201901‐0068, to Zhengbo Song). This study was sponsored by Zhejiang provincial program for the Cultivation of High‐level Innovative Health talents (to Zhengbo Song).

## CONFLICT OF INTEREST

All authors declare no conflict of interest.

## ETHICS APPROVAL AND CONSENT TO PARTICIPATE

Approval of the study protocol was obtained from Zhejiang Cancer Hospital Institutional Review Board Committee (approval number: IRB‐2022‐63). All patients' individual consent for this retrospective analysis was waived.

## CONSENT FOR PUBLICATION

Not applicable.

## Data Availability

The datasets used and/or analyzed during the current study are available from the corresponding author upon reasonable request.
